# Effects of long-term exposure to high altitude on brain structure in healthy people: an MRI-based systematic review and meta-analysis

**DOI:** 10.3389/fpsyt.2023.1196113

**Published:** 2023-06-26

**Authors:** Qiao Luo, Jie-Xin Zhang, Shuo Huang, Yong-He Hu, Han Wang, Xin Chen

**Affiliations:** ^1^Clinical Medical College, Chengdu University of Traditional Chinese Medicine, Chengdu, China; ^2^The Third People's Hospital of Chengdu City, Chengdu, China; ^3^Department of Laboratory Medicine, Southwest Jiaotong University, Chengdu, China; ^4^The General Hospital of Western Theater Command, Chengdu, China

**Keywords:** high altitude, MRI, brain structure, systematic review, meta-analysis

## Abstract

**Purpose:**

To conduct a systematic review and meta-analysis of observational studies of brain MRI, this paper assesses the effects of long-term exposure to high-altitude on brain structures in healthy people.

**Methods:**

Observational studies related to high-altitude, brain and MRI were systematically searched based on data retrieved from PubMed, Embase and Cochrane Library. The timescale for collecting literature was from the establishment of the databases to 2023. NoteExpress 3.2 was used to manage the literature. Two investigators performed literature screening and data extraction based on inclusion criteria, exclusion criteria, and literature quality. The quality of the literature was assessed using the NOS Scale. Finally, meta-analysis of included studies was performed using Reviewer Manager 5.3.

**Results:**

Initially, 3,626 articles were retrieved. After screening, 16 articles (*n* = 756 participants) were included in the systematic review, and meta-analysis was performed on 6 articles (*n* = 350 participants). The overall quality of the included articles was at medium level, with a mean NOS score of 5.62. The results of meta-analysis showed that the differences between the HA group and LA group were not statistically significant, in total GM volume (MD: −0.60, 95% CI: −16.78 to 15.58, *P* = 0.94), WM volume (MD: 3.05, 95% CI: −15.72 to 21.81, *P* = 0.75) and CSF volume (MD: 5.00, 95% CI: −11.10 to 21.09, *P* = 0.54).The differences between HA and LA in FA values of frontotemporal lobes were not statistically significant: right frontal lobe (MD: −0.02, 95% CI: −0.07 to 0.03, *P* = 0.38), left frontal lobe (MD: 0.01, 95% CI: −0.02 to 0.04, *P* = 0.65), right temporal lobe (MD: −0.00, 95% CI: −0.03 to 0.02, *P* = 0.78) and left temporal lobe (MD: −0.01, 95% CI: −0.04 to 0.02, *P* = 0.62). However, there were significant differences in GM volume, GM density and FA values in local brain regions between HA group and LA group.

**Conclusion:**

Compared with LA area, there were no significant differences in total GM, WM and CSF volumes in healthy people living at high-altitude area for long-term, while there were significant differences in GM volume and FA values in local brain regions. Long-term exposure to high-altitude area caused the adaptive structural changes in local brain regions. Since heterogeneity existed between the studies, further studies are needed to uncover the effects of high-altitude on brain of healthy people.

**Systematic review registration:**

https://www.crd.york.ac.uk/prospero/, identifier: CRD42023403491.

## 1. Introduction

The high-altitude environment is characterized by hypoxia, cold, dryness, intensive ultraviolet radiation and ecological fragility. While the percentage of oxygen in high altitude (HA) areas is ~21%, atmospheric pressure and oxygen pressure decrease with altitude. This results in low air pressure and oxygen deficiency, which may challenge the brain's adaptability (1). The brain is highly dependent on oxygen for its activity and is the most oxygen-consuming organ in the human body, accounting for about 20% of the body's oxygen intake (2). Long-term residence at HA areas causes structural changes in the brain, including cortical atrophy and high signal in the periventricular white matter (3). Even a single entry to a HA area causes microhemorrhages in the corpus callosum, suggesting a disruption of the blood-brain barrier (4). Residents who live in HA areas for a long time often experience memory storage, recall (5, 6), aphasia (7, 8) and attention deficits, and other functional impairments (9, 10) that suggest chronic damage to brain structures (11). Aside from direct effects, physiological adaptations during long-term exposure to HA, such as altered circulatory and respiratory function, can also lead to cumulative changes in the brain through afferent feedback. Thus, the high-altitude environment poses unique challenges to the brain's adaptability and can result in both structural and functional impairments (12).

Magnetic resonance imaging (MRI) is a widely-used non-invasive neuroimaging technique that offers excellent spatial resolution for the presentation and analysis of brain structure and function. Specifically, MRI effectively visualizes brain structure and evaluates various brain tissue types, including white matter (WM), gray matter (GM), cerebrospinal fluid (CSF), and regional brain volumes. Brain MRI analysis techniques use various methods to extract and interpret information from the collected data. For example, one such method is Amplitude of Low-Frequency Fluctuation (ALFF) analysis, which allows direct measurement of low-frequency neural oscillations during rest [as introduced by Biswal et al. (13)]. In addition, Regional Homogeneity (ReHo) analysis is another method used to measure brain connections between voxels and adjacent regions, as well as the synchronization of neuronal activity within specific brain regions (14). Another MRI technique, Diffusion Tensor Imaging (DTI), is based on the diffusion of water molecules in the brain to determine the microstructure, integrity and structure of WM, by measuring Fractional Anisotropy (FA) and Mean Diffusivity (MD) (15). Functional Connectivity (FC) analysis reveals a time-dependent, spatially distributed network of brain regions and connections, also known as the resting state network, by analyzing the activation time sequence data from two different types of neurons (16). Finally, Voxel-Mirrored Homotopic Connectivity (VMHC) analysis is another method that directly compares resting-state functional connectivity between the hemispheres by quantifying counterpart voxels between hemispheres (17).

With advances in MRI technology, researchers have increasingly turned to MRI to analyze the effects of high altitude on human brain structure and function. These investigations have uncovered the brain's compensatory processes in high-altitude environments. It has been found that long-term exposure to high altitude results in reduced gray matter (GM) density and damaged white matter (WM) fibers in various regions of the brain (6, 18, 19). Researchers studying soldiers stationed at high altitude observed significant increases in ALFF in several parts of the bilateral occipital cortex for the HA group, significant decreases in ALFF in the right anterior insula, and significant increases in functional connectivity (FC) in the right insula (20). Another study found that long-term exposure to high altitude reduced ReHo in specific brain regions, which was closely related to individual psychomotor disorders (21). In a study conducted by Zhang et al., FA values in various brain regions decreased significantly after mountaineering, suggesting that exposure to high altitude (6,206 m) can lead to disturbances in WM structure and integrity damage of fiber microstructure (22). Collectively, these findings suggest that individuals living in high-altitude areas exhibit unique brain function and morphology characteristics.

At present, it is uncertain what effect prolonged habitation in high-altitude environment has on the brain structure of people who are fully adapted to such conditions. Hence, this paper strives to conduct a systematic review and meta-analysis of observational studies that investigate the brain MRI characteristics of healthy people who have lived in high-altitude areas for prolonged periods. The aim is to provide a comprehensive evaluation of the effects of high-altitude conditions on brain structure and to determine whether there exist adaptive structural modifications in the brains of healthy people.

## 2. Materials and methods

### 2.1. Search strategy

The databases for literature retrieval were PubMed, Embase and Cochrane Library, and the timescale of retrieved literature was from the establishment of databases to February 2023. The language was limited to English, and the species was limited to human. The search terms were “high altitude/plateau/mountain/hypoxia,” “magnetic resonance/MRI,” and “brain/cerebrum/encephalon.” The complete search strategy was shown in Supplementary material.

### 2.2. Inclusion and exclusion criteria

Inclusion criteria were as follows: (1) observational studies, including cohort studies, cross-sectional studies and longitudinal studies; (2) high altitude (HA) group: long-term residents at high-altitude area, altitude > 2,500 m (23) or altitude > 1,500 m (24); low altitude (LA) group: long-term residents at low-altitude area, altitude < 2,500 m (23) or altitude < 1,500 m (24); (3) residence time >1 year; (4) the observation population are healthy people, age and gender not limited; (5) MRI outcomes of brain including WM, GM, CSF, FA, ReHo, FC, ALFF, VMHC, and so on.

Exclusion criteria were as follows: (1) non-observational studies, such as interventional studies, vitro experiments, and animal experiments; (2) inability to extract valid data from the articles; (3) articles with a too small sample size (*n* < 10); (4) duplicate literature; (5) full-text was not available; (6) participants combined with other causes of structural brain abnormalities: chronic mountain sickness, neurological disorders, history of previous head injury, abnormal brain development, brain vascular abnormalities, psychosomatic disorders, and structural brain changes caused by chronic poisoning.

### 2.3. Study screening and data extraction

The retrieved literature was managed by NoteExpress 3.0. Two investigators independently read the title, abstract, and full-text in sequence to screen the literature based on inclusion and exclusion criteria. In the case of disagreement, a third investigator would participate in a joint discussion to reach a consensus to determine whether to include the study.

Two investigators performed data extraction for the included studies according to the “Summarizing good practice guidelines for data extraction for systematic reviews and meta-analysis” (25), including the following information: first author, year of publication, country, region, study type, year of follow-up, age, sex, ethnicity and MRI outcomes, as well as data of altitude, duration of residence, duration of education, smoking, alcohol, and so on.

### 2.4. Quality assessment

The quality assessment of included studies was performed independently by two investigators. The quality of studies was assessed using the Newcastle-Ottawa Quality Assessment Scale (NOS) (26). The NOS included three aspects: study group selection (Selection), comparability between groups (Comparability), and outcomes of study (Outcome), with eight subscales and a total score of 9. The NOS scores for observational studies ranged from 1 to 9, with higher scores representing higher quality of study, and 0–3, 4–6, and 7–9 being categorized as low, medium, and high quality of study, respectively.

### 2.5. Statistical analysis

Continuous-type data were expressed by mean difference (MD) with 95% confidence interval, or standardized mean difference (SMD) with 95% confidence interval if the units of measurement for the outcome were inconsistent. According to heterogeneity, the meta-analysis chose either a fixed-effects model or a random-effects model to combine effect size. Heterogeneity of included studies was analyzed using chi-square test. If *I*^2^ < 50% and *P* > 0.1, heterogeneity between studies was considered within acceptable range, then the fixed-effects model was chosen. If *I*^2^ >50% or *P* < 0.1, heterogeneity between studies was considered high, then the random-effects model was chosen (27). If *P* < 0.05, the difference in the combined effect size of included studies was statistically significant. The above data were statistically analyzed by Review Manager 5.3.

## 3. Results

### 3.1. Study selection

**Figure 1:**
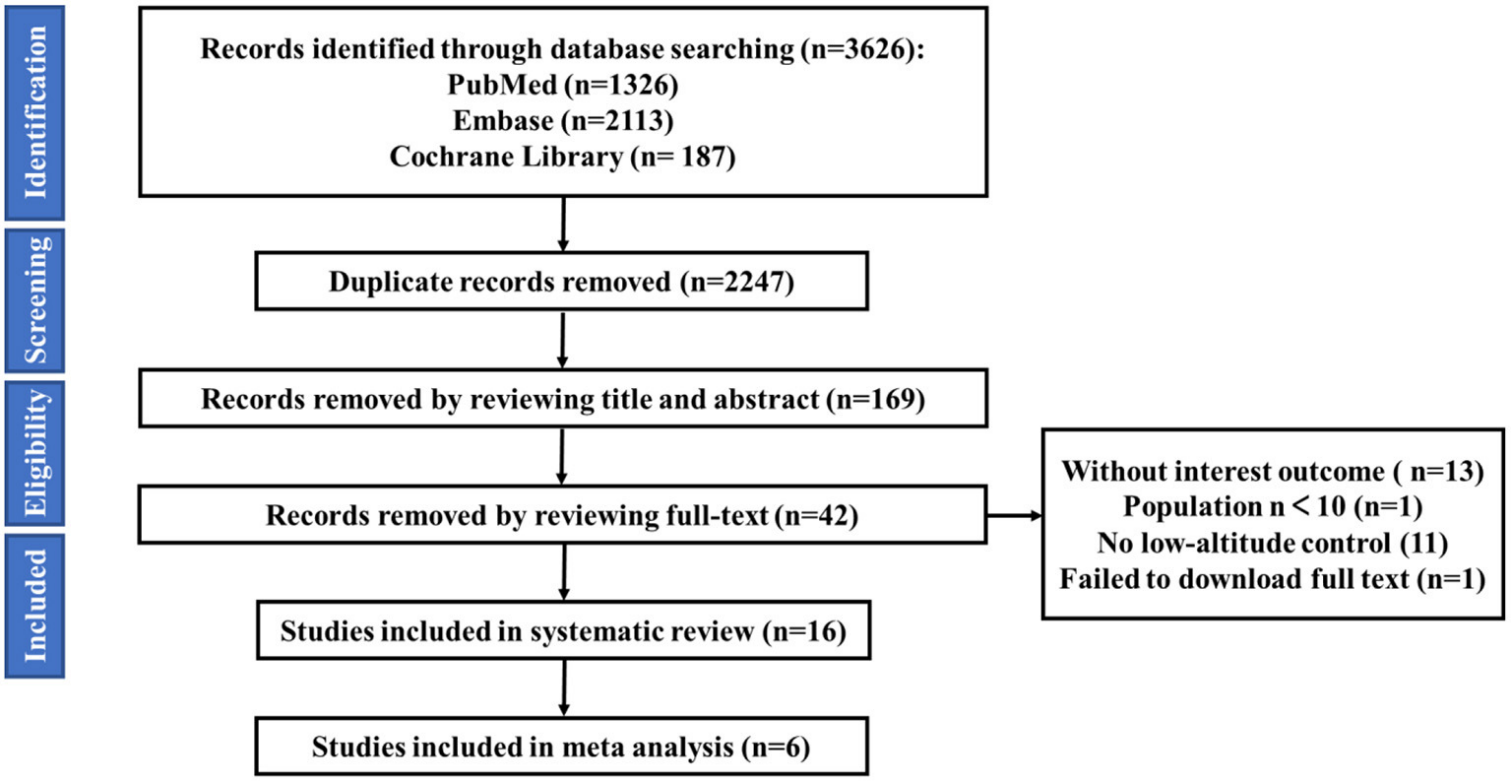
Literature search and screening process.

A total of 3,626 studies were retrieved, including 1,326 articles in PubMed, 2,113 articles in Embase, and 187 articles in Cochrane Library. Using NoteExpress 3.0 for literature management, the number of studies was reduced to 2,247 studies after removing duplicates. One hundred sixty-nine studies were screened by reading the title and abstract. According to the inclusion criteria, exclusion criteria and quality criteria, finally, 16 studies were included in the systematic review and 6 studies were included in the meta-analysis. The screening process is shown in below (Figure 1).

### 3.2. Quality assessment of eligible studies

Cohort studies with the total number of 13 studies were assessed via the Newcastle-Ottawa Scale (NOS). Eight studies had NOS scores of 6 (20, 28–34), and 5 studies scored 5 (35–39). The overall quality of included studies was at a medium level, with a mean NOS score of 5.62 (Table 1). Additional one cross-sectional study and two longitudinal studies were not assessed for quality.

**Table 1:** NOS scores of the included studies.

**References**	**Selection**	**Comparability**	**Outcome**	**NOS total score**
Zhang et al. (35)	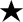	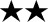	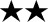	5
Zhang et al. (36)	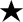	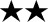	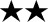	5
Zhong et al. (37)	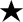	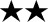	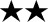	5
Zhang et al. (28)	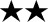	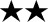	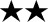	6
Chen et al. (34)	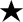	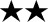	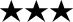	6
Wei et al. (29)	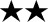	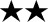	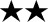	6
Chen et al. (38)	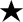	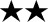	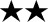	5
Zhang et al. (20)	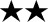	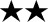	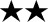	6
Chen et al. (38)	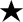	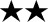	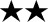	5
Chen et al. (30)	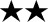	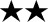	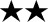	6
Zhang et al. (31)	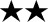	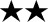	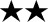	6
Zhang et al. (32)	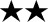	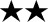	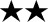	6
Zhang et al. (39)	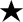	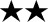	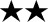	5
Yan et al. (33)	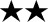	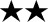	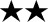	6

The 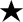, 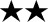, and 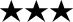 symbols indicate 1, 2, and 3 points, respectively.

### 3.3. Main characteristics of included studies

This systematic review included 16 observational studies, published between 2010 and 2023, and the main characteristics of the included studies are shown in Table 2. Thirteen of the 16 studies were cohort studies (20, 28–39), and one was a cross-sectional study (40), and two longitudinal studies (41, 42). Some of these studies were observations of different MRI outcomes in the same population [(41, 42) were the same population; (20, 30, 31, 38) were the same population; (33, 39) were the same population]. Excluding data from overlapping studies, with a total of 756 participants (418 cases in HA groups and 338 cases in LA groups). The number of participants in each study ranged from 32 to 157. The mean age of participants ranged from 14 to 48 years old, participants of 10 studies were adults (51.59%) and 2 studies were adolescents (34.54%), and 3 studies included both adults and adolescents (14–20 years old, 22.09%). The mean duration of education ranged from 6 to 15 years. Ten studies reported smoking consumption, of which 9 studies were non-smokers. Two studies reported alcohol consumption, of which 1 study was non-drinkers.

**Table 2:** Basic characteristics of included studies.

**References**	**Subjects, (*n*)**	**HA/LA, (*n*)**	**Gender, M (F)**	**Age (year), mean ±SD**	**Education (year), mean ±SD**	**Smoking, (*n*)**	**Alcohol, (*n*)**
Zhang et al. (35)	45	24/21	10 (14)/10 (11)	21.1 ± 1.2/20.4 ± 0.8	14.7 ± 0.4/14.6 ± 0.4	0/0	NA
Zhang et al. (36)	45	25/20	11 (14)/10 (10)	21.0 ± 1.2/20.6 ± 1.4	College students	NA	NA
Zhong et al. (37)	54	27/27	11 (16)/13 (14)	38.6 ± 5.4/41.7 ± 6.4	12.0 ± 0.6/12.5 ± 3.0	NA	NA
Zhang et al. (28)	92	49/43	20 (29)/19 (24)	30.9 ± 6.0/32.9 ± 6.8	14.8 ± 1.9/15.1 ± 1.7	14/15	11/14
Feng et al. (40)	66	33/33	33 (0)/33 (0)	31.9 ± 4.7/31.9 ± 5.1	15.8 (12–17)/NA	0/0	0/0
Xin et al. (41)	69	69/NA	48 (21)/NA	18.2 ± 0.3/NA	College freshman	0/0	NA
Chen et al. (34)	98	49/49	32 (17)/32 (17)	17–20/17–20	College freshman	0/0	NA
Zhang et al. (20)	32	16/16	16 (0)/16 (0)	20.5 ± 0.7/19.9 ± 1.5	6.7 ± 3.9/7.5 ± 5.0	0/0	NA
Wei et al. (29)	157	77/80	34 (43)/34 (46)	14–18/14–18	Middle school students	NA	NA
Chen et al. (42)	69	69/NA	48 (21)/NA	18.2 ± 0.3/NA	College freshman	0/0	NA
Zhang et al. (20)	32	16/16	16 (0)/16 (0)	20.5 ± 0.7/19.9 ± 1.5	6.7 ± 3.9/7.5 ± 5.0	0/0	NA
Chen et al. (38)	32	16/16	16 (0)/16 (0)	20.5 ± 0.7/19.9 ± 1.5	6.7 ± 3.9/7.5 ± 5.0	0/0	NA
Chen et al. (30)	32	16/16	16 (0)/16 (0)	20.5 ± 0.7/19.9 ± 1.5	6.7 ± 3.9/7.5 ± 5.0	0/0	NA
Zhang et al. (31)	32	16/16	16 (0)/16 (0)	20.5 ± 0.7/19.9 ± 1.5	6.7 ± 3.9/7.5 ± 5.0	0/0	NA
Zhang et al. (32)	42	21/21	6 (15)/6 (15)	16.3 ± 0.8/16.5 ± 0.7	High school student	NA	NA
Zhang et al. (39)	56	28/28	12 (16)/12 (16)	20.4 ± 1.4/20.9 ± 1.5	13.3 ± 0.8/13.4 ± 0.6	NA	NA
Yan et al. (33)	56	28/28	12 (16)/12 (16)	20.4 ± 1.4/20.9 ± 1.5	13.3 ± 0.8/13.4 ± 0.6	NA	NA

All included studies were conducted in China, with the HA groups reside in the Tibetan plateau or Lhasa, and the LA groups reside in Xiamen, Minhe County, Chengdu or other sea level city. The altitude of HA groups ranged from 2,300 to 5,300 m, while the altitude of LA groups all below 2,500 m. Ten studies were Han, 1 study was Tibetan and 5 studies were Tibetan in the HA group and Han in the LA group. Participants of 9 studies were between 2 and 4 years of residence, and 7 studies were born and raised in the HA area (19–48 years). In MRI outcomes, GM (*n* = 11), WM (*n* = 5), CSF (*n* = 5), FA (*n* = 7), and Cortical thickness (*n* = 2) were structural brain indicators, and ReHo (*n* = 5), ALFF (*n* = 4), FC (*n* = 5) and VMHC (*n* = 2) were functional brain indicators. In this review, only the structural brain indicators were analyzed. The altitudinal environmental characteristics and MRI Outcomes included in the review are shown in Table 3.

**Table 3:** Altitudinal environmental characteristics and MRI outcomes of the included studies.

**References**	**Ethnic group**	**Living area, HA/LA**	**Altitude (m)**	**Residence time**	**MRI outcomes**
Zhang et al. (35)	Tibetan/Han	Qinghai-Tibet Plateau/Xiamen	3,600–4,200/ < 500	Born and raised	Cortical thickness, FC
Zhang et al. (36)	Tibetan/Han	Lhasa/sea level city	3,658/sea-level	Born and raised	GM, ALFF, ReHo, VMHC, FC
Zhong et al. (37)	Tibetan/Han	Qinghai-Tibet Plateau/sea level city	>2,800/ < 500	Born and raised	GM, ALFF
Zhang et al. (28)	Tibetan/Han	Qinghai-Tibet Plateau/Minhe County	4,300/1,700	Born and raised	GM, FA, ReHo, ALFF
Feng et al. (40)	Han/Han	Lhasa/sea level city	3,658/sea-level	2 years	GM, WM, CSF
Xin et al. (41)	Han/NA	Lhasa/NA	3,658/NA	2 years	FC
Chen et al. (34)	Han/Han	Lhasa/sea level city	3,658/sea-level	2 years	GM, FA
Wei et al. (29)	Tibetan/Han	Qinghai-Tibet Plateau/sea level city	2,300–5,300/sea level	Born and raised	Cortical thickness
Chen et al. (42)	Han/NA	Lhasa/NA	3,658/NA	2 years	GM, ReHo, FC
Zhang et al. (20)	Han/Han	Qinghai-Tibet Plateau/Xiamen	2,300–4,400/ < 100	2 years	ALFF, FC
Chen et al. (38)	Han/Han	Qinghai-Tibet Plateau/sea level city	2,300–4,400/sea level	2 years	VMHC, FA
Chen et al. (30)	Han/Han	Qinghai-Tibet Plateau/sea level city	2,300–4,400/sea level	2 years	Reho
Zhang et al. (31)	Han/Han	Qinghai-Tibet Plateau/Xiamen	2,300–4,400/ < 100	2 years	GM, WM, CSF, FA
Zhang et al. (32)	Tibetan/Tibetan	Qinghai-Tibet Plateau/Xiamen	2,900–4,700/ < 400	4 years	GM, WM, CSF, FA, MD
Zhang et al. (39)	Han/Han	Qinghai-Tibet Plateau/Chengdu	2,616–4,200/ < 400	Born and raised	GM, WM, CSF, FA
Yan et al. (33)	Han/Han	Qinghai-Tibet Plateau/sea level city	2,616–4,200/ < 400	Born and raised	GM, WM, CSF, FA, ReHo

### 3.4. Main outcomes and meta-analysis

#### 3.4.1. GM

Eleven studies (28, 31–37, 39, 40, 42) reported GM. Five studies reported total GM volume (31–33, 39, 40), and 5 studies reported only regional GM volume (34–37, 42), and 1 study reported GM density (28).

Zhang et al. found that the GM volume and cortical thickness of the left somatosensory and motor cortex, left second visual cortex and right visual cortex were significantly increased in Tibetans living at high altitude for a long time (>19 years) compared with lowlanders (35). Another study by the same team found that Tibetans who lived at high altitudes for a long time had increased GM volume in the visual cortex, hippocampus and rectus (36). Another study showed that the GM volume in HA residents is significantly higher than LA residents in the bilateral somatosensory cortex and vision cortex (37). Zhang et al. observed medical personnel in Maduo County (HA group, 4,300 m) and Minhe county (LA group, 1,700 m), the HA group had significantly lower GM density of the left olfactory cortex, right medial orbital superior frontal gyrus, bilateral insula, left globus pallidus, and temporal lobe (28). Feng et al.'s observation of healthy people who migrated to Lhasa (3,658 m) for 2 years, showed that no significant change in total GM volume compared with the SL group, but there were decreased regional GM volume in the HA group, such as putamen, insula, amygdala, pale nucleus, hippocampus, amygdala (40). A longitudinal observation of 49 Tibetan university freshmen (HA group) from a low altitude area [Xi'an (466 m), China] showed that after 2 years of high-altitude exposure, bilateral caudate thickness significantly decreased, while GM volumes of bilateral thalamus, putamen, hippocampus and amygdala did not change significantly (34). Another study followed 69 university freshmen who immigrated to Tibet, found a significant decrease in GM volume in the left cisternal nucleus (42). Zhang et al. found that, after migrating to the Tibetan Plateau for 2 years, GM density was significantly increased in some brain regions (right postcentral gyrus and right superior frontal gyrus) and significantly decreased in other brain regions (right middle frontal gyrus, right parahippocampal gyrus, right inferior middle temporal gyrus, etc.) (31). A study concerning Tibetan adolescents born and raised on the Tibetan plateau, found that no significant difference in total GM volume, but a significant decrease in regions GM volume (left insula, left inferior parietal gyrus, and right superior parietal gyrus), compared with indigenous residents in low-altitude area (32). An observation of Han adolescents, born and raised on the Tibetan Plateau, found reduced GM volumes in the bilateral anterior insula, bilateral prefrontal cortex, left precentral gyrus, left cingulate gyrus, and right lingual cortex (33).

**Figure 2:**
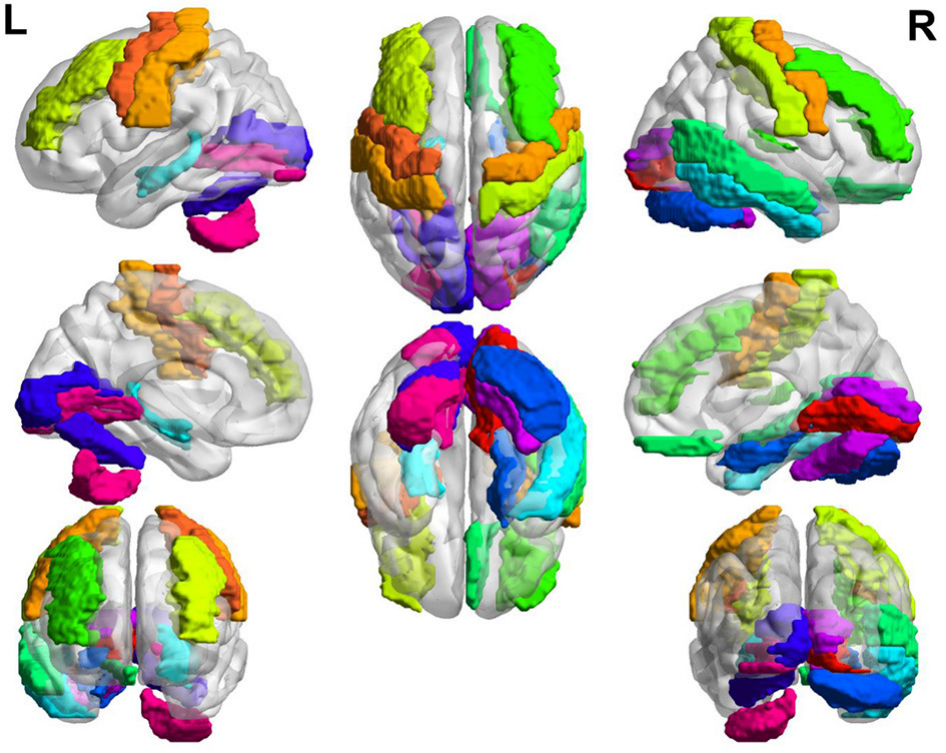
Brain regions with increased GM volume.

**Figure 3:**
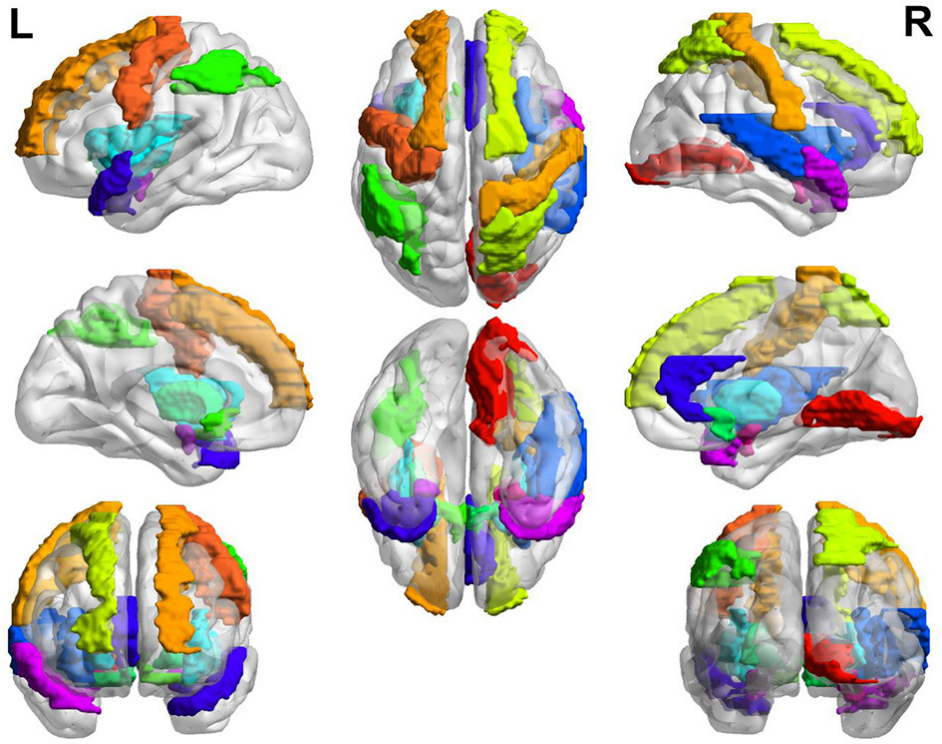
Brain regions with decreased GM volume.

In general, the brain regions with increased GM volume after long-term exposure to high altitude are mainly distributed in left somatosensory, motor cortex, auditory cortex, visual cortex, calcarine sulcus, lingual gyrus, hippocampus, rectus, postcentral, middle frontal gyrus, parahippocampal gyrus, and so on. The brain regions with increased GM volume are mainly distributed in putamen, insula, amygdala, olfactory, hippocampus, temporal pole, pallidum, inferior parietal gyrus, superior parietal gyrus, anterior cingulate cortex, prefrontal cortex, precentral cortex, and so on. The overall view of the brain regions with increased GM volume in HA group is shown in Figure 2 and the overall view of the brain regions with decreased GM volume in HA group is shown in Figure 3.

**Figure 4:**
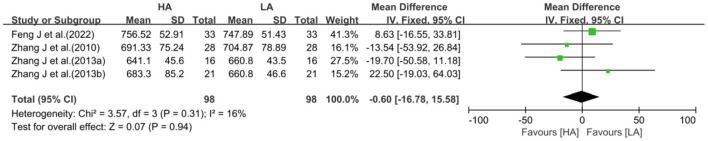
Meta-analysis of the effect of HA and LA on GM volume.

Four studies (31, 32, 39, 40) reported total GM volume in HA group and LA group (*n* = 196), and meta-analysis of outcomes was performed. The test for heterogeneity (*I*^2^ = 16%, *P* = 0.31) suggested low between-study heterogeneity, so a fixed-effects model was used. Meta-analysis showed no statistically difference in the effect of HA and LA on total GM volume (MD: −0.60, 95% CI: −16.78 to 15.58, *P* = 0.94; Figure 4).

#### 3.4.2. WM

Four studies (31, 32, 39, 40) reported total WM volume in the brain of HA group and LA group (*n* = 196). Feng et al.'s observation of 33 high-altitude residents and 33 sea level residents found that total WM volume was significantly higher in the HA group compared to the LA group (40). Zhang et al.'s observation of 16 Han male military personnel who had migrated to high-altitude for 4 years found no significant difference in total WM volume compared to LA group (31). An observation of 21 Tibetan adolescent students (HA group) showed no significant differences in total WM volume compared to LA group (32), and another observation of 28 Han adolescents (HA group) living on the Tibetan plateau also showed no significant differences in total WM volume compared to LA group (39).

**Figure 5:**
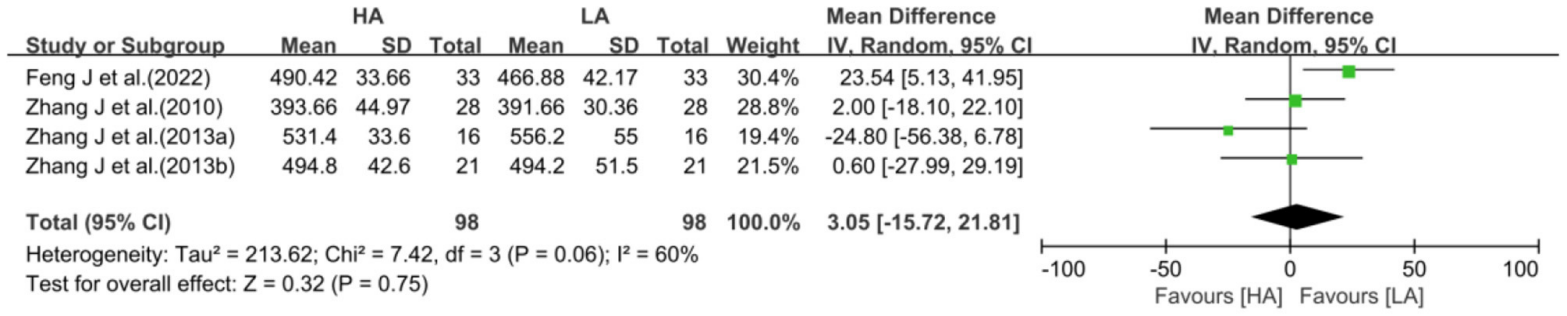
Meta-analysis of the effect of HA and LA on WM volume.

Four studies with a test of heterogeneity (*I*^2^ = 60%, *P* = 0.06) suggested a high degree of between-study heterogeneity, and a random-effects model was selected for meta-analysis. Meta-analysis showed no statistically difference in the effect of HA and LA on total WM volume (MD: 3.05, 95% CI: −15.72 to 21.81, *P* = 0.75; Figure 5). After excluding 1 study (40), the between-study heterogeneity was significantly reduced (*I*^2^ = 5%, *P* = 0.35), and meta-analysis showed no statistical difference between the effects of HA and LA on total WM volume (*P* = 0.58).

#### 3.4.3. CSF

In the included studies, 4 studies (31, 32, 39, 40) reported total CSF volume in the brains of HA group and LA group (*n* = 196). Feng et al.'s observational study showed no significant difference in total CSF volume between the HA group and LA group (40). Zhang et al.'s observation of 16 Han male military personnel who had migrated to high altitude for 4 years, showed no significant difference in total CSF volume compared to LA group (31). An observation of 21 Tibetan adolescent students also showed no significant difference in total CSF volume compared to LA group (32). While an observation of 28 Han adolescents indigenous to the Tibetan Plateau showed a significant increase in total CSF volume (39).

**Figure 6:**
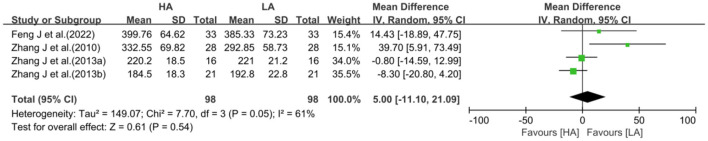
Meta-analysis of the effect of HA and LA on CSF volume.

With a high degree of heterogeneity among the 4 studies (*I*^2^ = 61%, *P* = 0.05), a random-effects model was selected for meta-analysis. Meta-analysis showed no statistically difference in the effect of HA and LA on total CSF volume (MD: 5.00, 95% CI: −11.10 to 21.09, *P* = 0.54; Figure 6). After excluding 1 study (39), the between-study heterogeneity was significantly decreased (*I*^2^ = 0%, *P* = 0.40), and meta-analysis suggested that there was no statistical difference between the effects of HA and LA on total CSF volume (*P* = 0.44).

#### 3.4.4. FA

In the included studies, 7 studies (28, 31–34, 38, 39) reported FA values of regional WM [(31, 38) are the same population; (33, 39) are the same population]. Zhang et al. observed medical personnel in Maduo County (HA group, 4,300 m) and Minhe county (LA group, 1,700 m) found that FA values were significantly reduced in the corpus callosum, bilateral radial corpuscles and left internal capsule area in the HA group compared to the LA group (28). A longitudinal observation of 49 Tibetan University freshmen (HA group) after 2 years of high-altitude exposure showed increased FA values in the right posterior corona radiate and splenium of corpus callosum, and decreased FA values in the superior longitudinal fasciculus, right anterior limb of internal capsule and body of corpus callosum (34). In the HA group, Chen et al. found that the FA value of the fibers connecting the bilateral visual cortex was significantly reduced in the spleen of the corpus callosum, suggested that there may be an important neural mechanism of visual dysfunction (38). Zhang et al. found that FA values were significantly reduced in the corpus callosum, paracentral lobule, and bilateral hippocampus, while FA values were significantly increased in the frontal lobe, inferior temporal gyrus, and internal capsule, in the HA group compared to LA group (31). Another study of adolescents by the same team showed that the HA group had lower FA values in a wide range of brain regions (frontal, temporal, insula, thalamus, midbrain regions, and so on) (32). Yan et al.'s study showed that HA residents had significantly lower FA values in the right posterior cingulate and right precentral cortex, and significantly higher FA values in the right and left anterior limbs of the internal capsule (33).

**Figure 7:**
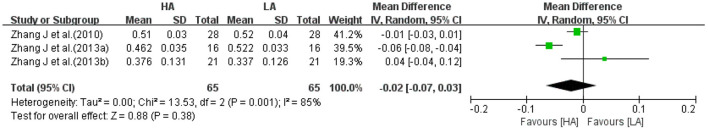
Meta-analysis of the effect of HA and LA on FA values in the right frontal lobe.

Three studies (31, 32, 39) reported right frontal FA values for HA group and LA group (*n* = 130). The heterogeneity test (*I*^2^ = 85%, *P* = 0.001) suggested a high degree of between-study heterogeneity, so a random-effects model was selected for analysis. Meta-analysis showed no statistically difference in the effect of HA and LA on right frontal FA values (MD: −0.02, 95% CI: −0.07 to 0.03, *P* = 0.38; Figure 7). One of the studies (31) was all male participants, and heterogeneity was significantly reduced after excluding the study (*I*^2^ = 31%, *P* = 0.23), then chose a fixed-effects model for analysis. The combined effect sizes of studies suggested that the effect of long-term exposure to high-altitude on right frontal FA values was not statistically significant (*P* = 0.42).

**Figure 8:**
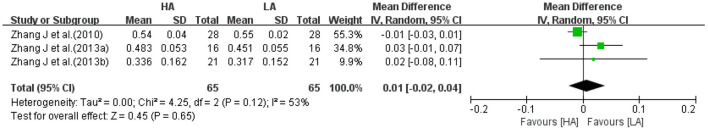
Meta-analysis of the effect of HA and LA on FA values in the left frontal lobe.

Three studies (31, 32, 39) reported left frontal FA values for HA group and LA group (*n* = 130). The test for heterogeneity (*I*^2^ = 53%, *P* = 0.12) suggested a high degree of between-study heterogeneity, so a random-effects model was selected for analysis. Meta-analysis showed no statistically difference in the effect of HA and LA on left frontal FA values (MD: 0.01, 95% CI: −0.02 to 0.04, *P* = 0.65; Figure 8). After excluding a single-sex study (31), heterogeneity was significantly reduced (*I*^2^ = 0%, *P* = 0.56), and the results of combined effect size suggested that the effect of long-term exposure to high-altitude on left frontal FA values was not statistically significant (*P* = 0.27).

**Figure 9:**
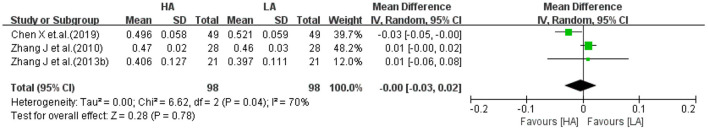
Meta-analysis of the effect of HA and LA on FA values in the right temporal lobe.

Three studies (32, 34, 39) reported FA values of right temporal lobe for HA group and LA group (*n* = 196). The heterogeneity test of the included studies (*I*^2^ = 70%, *P* = 0.04) suggested high inter-study heterogeneity, so a random-effects model was used. Meta-analysis showed that the effect of long-term exposure to high-altitude on right temporal lobe FA values was not statistically significant (MD: −0.00, 95% CI: −0.03 to 0.02, *P* = 0.78; Figure 9).

**Figure 10:**
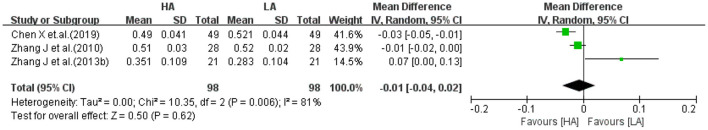
Meta-analysis of the effect of HA and LA on FA values in the left temporal lobe.

Three studies (32, 34, 39) reported FA values of left temporal lobe for HA group and LA group (*n* = 196). The heterogeneity test (*I*^2^ = 81%, *P* = 0.006) suggested a high degree of heterogeneity between studies, so a random-effects model was selected for analysis. Meta-analysis showed that the effect of long-term exposure to high-altitude on left temporal FA values was not statistically significant (MD: −0.01, 95% CI: −0.04 to 0.02, *P* = 0.62; Figure 10).

#### 3.4.5. Subgroup analysis

**Figure 11:**
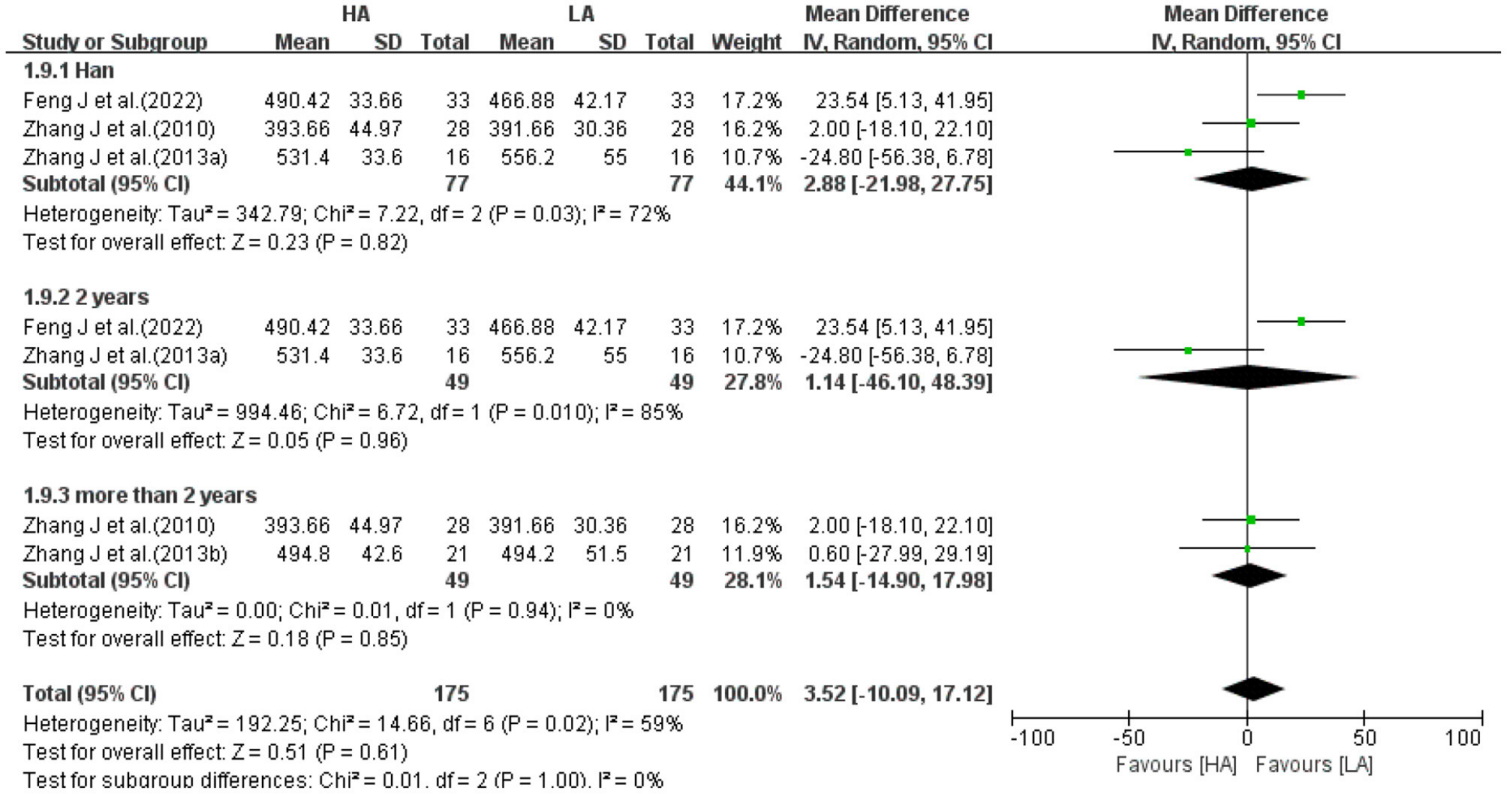
Subgroup analysis of the effect of HA and LA on WM volume.

**Figure 12:**
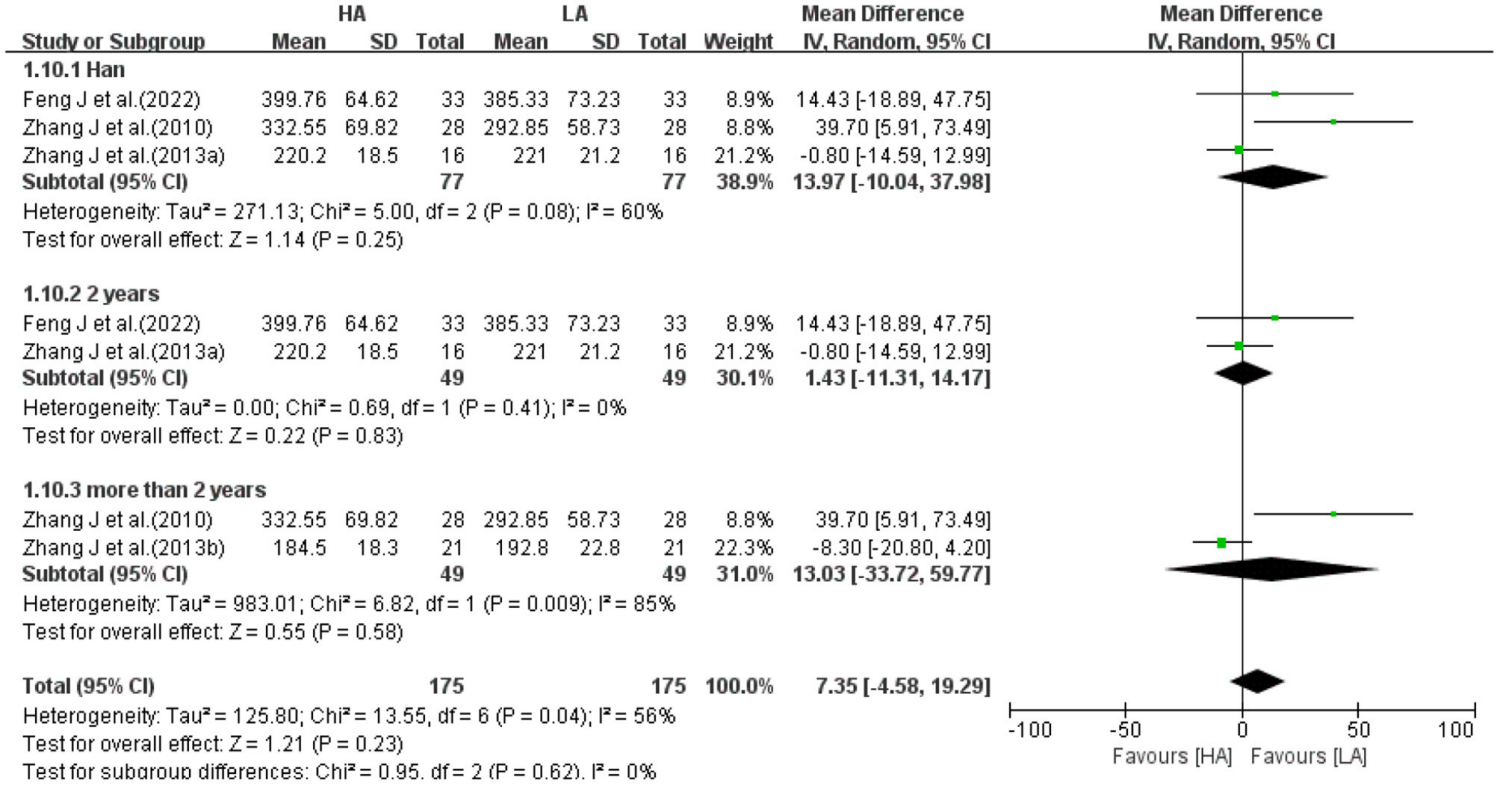
Subgroup analysis of the effect of HA and LA on CSF volume.

Because heterogeneity among some studies were high, subgroup analysis was performed for 2 volume indicators (WM, Figure 11; CSF, Figure 12). Excluding 1 study of Tibetans, analyzing 3 studies of Han, the results suggested no statistical difference between the effects of HA and LA on WM (*P* = 0.82) and CSF (*P* = 0.25). Grouping the studies according to the duration of high-altitude exposure, there were no statistical difference between HA group and LA group on WM (*P* = 0.96) and CSF (*P* = 0.83) for 2 years high-altitude exposure, and no statistical difference between HA group and LA group on WM (*P* = 0.85) and CSF (*P* = 0.58) for more than 2 years high-altitude exposure. The number of studies on FA values was too small to allow subgroup analysis of frontotemporal FA values based on elements such as age, gender, ethnicity, altitude and duration of residence.

## 4. Discussion

The effects of long-term exposure to high-altitude on brain structures in people with altitude sickness are now well established. People with altitude sickness entering high-altitude area may occur cerebral edema (43, 44), cerebral hemorrhage (45–47), disruption of the integrity of the blood-brain barrier (48, 49), brain atrophy (50–52), and other structural changes in the brain. What about healthy people who are well adapted to the high-altitude environment? Do structural brain changes also occur? To our knowledge, this is the first study using systematic review to analyze the association between brain structure and high-altitude exposure in a healthy people.

### 4.1. Summary of included studies

The 16 included studies were all observational studies of healthy people living at high-altitude area, excluding duplicate data, with a total of 756 participants (418 in the HA group and 338 in the LA group). GM, WM and CSF were the major structures that made up the brain, and 5 studies (31–33, 39, 40) reported the total volume of GM, WM and CSF. Meta-analysis showed that the differences between HA and LA in GM volume (*P* = 0.94), WM volume (*P* = 0.75) and CSF volume (*P* = 0.54) were not statistically significant. Three studies (31, 32, 39) reported frontal FA values and 3 studies (32, 34, 39) reported temporal lobe FA values. Meta-analysis showed no statistically significant differences between HA group and LA group in the right frontal lobe (*P* = 0.38), left frontal lobe (*P* = 0.65), right temporal lobe (*P* = 0.78) and left temporal lobe (*P* = 0.62). Subgroup analysis of 2 brain structure (WM, CSF) volumes with high heterogeneity, removing the effects of ethnicity and the exposure duration of high-altitude, the differences in the effects of HA group and LA group on WM volume and CSF volume were not statistically different (*P* > 0.05). Therefore, it can be concluded that long-term exposure to high-altitude had no significant effect on total volume of GM, WM, CSF, and FA values of frontotemporal lobe in healthy people. However, various studies showed that long-term exposure to high-altitude had significant effects on GM volume, GM density, and FA values in local brain regions.

### 4.2. GM and HA

Gray matter (GM) is formed by a large number of neuronal cell bodies and their dendrites aggregated together, covering the surface of the cerebral hemispheres and cerebellum as well as the exterior of the brainstem. GM is a major component of functional brain areas, such as the frontal, temporal, parietal, occipital lobes, basal ganglia, and cerebellum. The frontal lobe plays a central role in most aspects of cognition, decision making progress and execution, and it is responsible for reasoning, learning and creativity (53). It is critical for the execution of complex tasks such as goal-driven, problem solving, self-awareness and emotion regulation (54). Studies have found that GM volume of frontal lobe changes are seen in psychiatric disorders such as cognitive impairment, dementia (55) and schizophrenia (56). The temporal lobe is responsible for brain functions such as learning (57), long-term memory, working memory (58), cognition, spatial information processing and creative association (59). It was found that GM volume of temporal lobe was associated with psychological stress (6), social anxiety (60), depression (61, 62) and schizophrenia (63, 64) and other psychiatric disorders. The cerebellum is involved in the coordination or integration of motor, sensory, and cognitive processes (65), and during adolescence, the GM volume of the cerebellum is associated with attention (66), sleep (67), and cognitive function (68).

In the included studies, 11 studies reported GM, 5 studies with 2–4 years of high-altitude exposure and 6 studies with more than 10 years of high-altitude exposure. Two years of high-altitude exposure resulted in significant reduction in brain volume in the caudate nucleus and left putamen (34, 42), while GM in the thalamus, hippocampus and amygdala did not change significantly. The involvement of the caudate artery in chronic hypoxic diseases such as obstructive sleep apnea and chronic obstructive pulmonary disease has been demonstrated (69, 70). Sensitivity to hypoxia (71) suggested that hypoxia in high-altitude environments may induce caudate artery constriction, leading to ischemic atrophy of the caudate nucleus. Putamen is associated with working memory, cognitive flexibility and attention (72). Previous studies have confirmed that the left-shell nucleus is more prone to hypoxia than the right-shell nucleus (73), particularly chronic sustained hypoxia. Exposure to high-altitude for a shorter period of time (2–4 years) first caused GM atrophy in brain regions sensitive to ischemia and hypoxia, such as left-shell nucleus.

Whereas, studies (32, 35, 36, 39) of exposure to high-altitude for longer time (17–40 years) showed increased or decreased GM volumes in a wider range of brain regions. These studies showed a significant decrease in GM of frontotemporal lobes closely linked to cognition and a significant increase of GM in brain regions related to motor, sensory and visual. Studies have confirmed that prolonged exposure to high-altitude impaired cognitive functions such as attention, memory, and inhibitory control (74), and the reduction of frontotemporal may be the structural basis of cognitive impairment. In addition, there were studies confirmed that hypoxia and low pressure impair standing balance, which is mainly regulated and integrated by brain regions of sensory, motor, visual and vestibular (75). In studies of prolonged exposure (17–40 years), healthy people in high-altitude area showed an adaptive enlargement in these brain regions.

### 4.3. FA and HA

FA is the fiber diameter and density, reflecting the microstructural characteristics of WM fibers. A decrease in FA value implies a disruption of WM structure (76). Disruption of cerebral WM structures can lead to hippocampal atrophy (77), inducing cognitive impairment and dementia (78). These WM structural disruptions are mainly caused by cerebral small vessel disease (79). Age-related structural disruption of WM is associated with cognitive decline in a healthy elderly people (80). In the review, 7 studies reported FA values, and meta-analysis showed that the effect on FA values in the frontotemporal lobe was not statistically significant. Three studies reported reduced FA values in the corpus callosum. The corpus callosum is the most important connection between the cortical areas of the brain in both hemispheres, connecting the fibers of motor, sensory and visual cortices (81). Studies have found that patients with acute plateau brain edema may present with multiple microhemorrhages along the corpus callosum (46, 82) and iron heme deposits (83). In addition, transient bilateral visual loss associated with the corpus callosum can occur in people who acutely enter high-altitude areas (84). Studies above suggested that the corpus callosum is a brain region sensitive to high-altitude environment.

### 4.4. Brain function and HA

A total of 9 studies (20, 28, 30, 35–38, 41, 42) examined the impact of high altitude on brain function, using functional indicators such as ReHo, ALFF, FC, and VMHC (see Table 3). The results indicated that prolonged exposure to high altitude might have different effects on brain function. In terms of ReHo analysis, the included studies produced inconsistent results. Zhang et al. found no significant difference in ReHo values between high-altitude HA group and LA group (28, 36). However, Chen et al. reported ReHo increased significantly in bilateral hippocampus, and decreased significantly in bilateral putamen, bilateral superior temporal gyrus, and medial frontal gyrus, after prolonged exposure to high-altitude (42). Chen et al. reported a significant increase in global mean ReHo in the HA group, as well as a regional increase in the right inferolateral sensorimotor cortex (30). ALFF was used to evaluate the amplitude of local spontaneous neuronal activity. Zhang et al. observed an increase in ALFF values in the left putamen and left fusiform gyrus, while Zhang et al. observed decreased ALFF values in the left cerebellum, left putamen, left orbital inferior frontal gyrus, and left precuneus in the HA group (28, 35). Zhang et al. reported significant increases of ALFF in several sites within the bilateral occipital cortices, while showing significant decreases of ALFF in the right anterior insula in the HA group (20). FC analyzed the correlation of neural activity in different brain regions by analyzing the activation of two different neurons in the brain. Xin et al. and Chen et al. analyzed whole-brain functional networks, which revealed that participants presented reduced FC after exposure to high-altitude in bilateral superior temporal gyrus, bilateral striatum, bilateral postcentral gyrus, cingulate gyrus, medial frontal gyrus, right cerebellum, right hippocampus, and right brainstem (41, 42). In Zhang et al.'s study, HA group showed an increase FC in right insular, right superior temporal gyrus, right posterior central gyrus, Loran's gyrus, superior limbic gyrus, and subfrontal trigone (20). VMHC was a method used to examine resting-state functional connectivity between hemispheres. Compared with the LA group, HA group had significantly higher VMHC in the bilateral visual cortex (38) and lower VMHC values in the precentral gyrus (36). The results of these included studies suggest that long-term exposure to high-altitude has a wide range of effects on brain function. However, the relationship between functional changes and structural changes needs to be revealed by subsequent studies.

### 4.5. Limitations

There were some limitations to this systematic review. Firstly, the total number of studies was low and sample sizes of some studies were small. Secondly, the overall quality of studies was low and there was a lack of high-quality studies. Thirdly, there was a high degree of heterogeneity among studies for the following reasons. Data on follow-up, smoking, alcohol and duration of education were incomplete. In some included studies, the time span of inter-study observations is large-ranging from 2 years to over 40 years. Moreover, HA group of included studies were conducted on the Tibetan plateau, which lead to geographical limitations, thus, could not fully represent the environmental characteristics of other high-altitude areas. Finally, some of the study populations were students, military and medical personnel, which were specific populations and not representative of the overall population characteristics.

## 5. Conclusions

Compared with the residents in the LA area, there were no significant differences in total GM, WM and CSF volumes in healthy people living at high-altitude area for long period of time, while there were significant differences in GM volume and FA values in local brain regions. Long-term exposure to the high-altitude environment caused adaptive structural changes in the local brain regions. In the end, since heterogeneity existed between the studies, further studies are needed to uncover the effects of high-altitude on brain of healthy people.

## Data availability statement

The original contributions presented in the study are included in the article/Supplementary material, further inquiries can be directed to the corresponding authors.

## Author contributions

XC conceived and designed the study and provided funding support. QL performed the data collection and analysis. SH contributed to the literature retrieval. J-XZ visualized and tabulated the data. HW and Y-HH reviewed and refined the paper. All authors contributed to the paper and approved the submitted version.
