# Supplementary material for: Effects of long-term exposure to high altitude on brain structure in healthy people: an MRI-based systematic review and meta-analysis
Source: Front Psychiatry. 2023 Jun 26;14:1196113. doi: 10.3389/fpsyt.2023.1196113 (PMC10330765; doi:10.3389/fpsyt.2023.1196113)
Supplement: Supplementary file 1 [file Data_Sheet_1.docx]

Supplementary Material

Effects of long-term exposure to high altitude on brain structure in healthy people: an MRI-based systematic review and meta-analysis

Qiao Luo^1†^, Jie-Xin Zhang^3^, Shuo Huang^1^, Yong-He hu^4^, Han Wang^2*^, Xin Chen^2*^

*** Correspondence:**Xin Chen
[xinchencd@yeah.net](mailto:xinchencd@yeah.net)

Han Wang

[wanghan@swjtu.edu.cn](mailto:wanghan@swjtu.edu.cn)

Xin Chen^*^ and Han Wang^*^ are Co-corresponding authors.

Qiao Luo^†^ is the first author.

# Supplementary Data

# Search strategies for all databases

# 1. Search Strategy for PubMed:

# #1 high altitude[Title/Abstract] OR plateau[Title/Abstract] OR mountain[Title/Abstract] OR hypoxia[Title/Abstract] (213105)

# #2 magnetic resonance[Title/Abstract] OR MRI[Title/Abstract] (588238)

# #3 brain [Title/Abstract] OR cerebrum [Title/Abstract] OR encephalon [Title/Abstract]

# #4 #1 and #2 and #3 (1326)

# 2. Search Strategy for Cochrane library:

# #1 (high altitude OR plateau OR mountain OR hypoxia):ti,ab,kw(101334)

# #2 (magnetic resonance OR MRI):ti,ab,kw(45427)

# #3 (brain OR cerebrum OR encephalon):ti,ab,kw (72485)

# #4 #1 and #2 and #3 (187)

# 3. Search Strategy for Embase:

# #1 'high altitude':ab,ti OR plateau:ab,ti OR mountain:ab,ti OR hypoxia:ab,ti (270676)

# #2 'magnetic resonance':ab,ti OR mri:ab,ti (835682)

# #3 brain:ab,ti OR cerebrum:ab,ti OR encephalon:ab,ti (1516500)

# #4 #1 and #2 and #3 (2113)

**
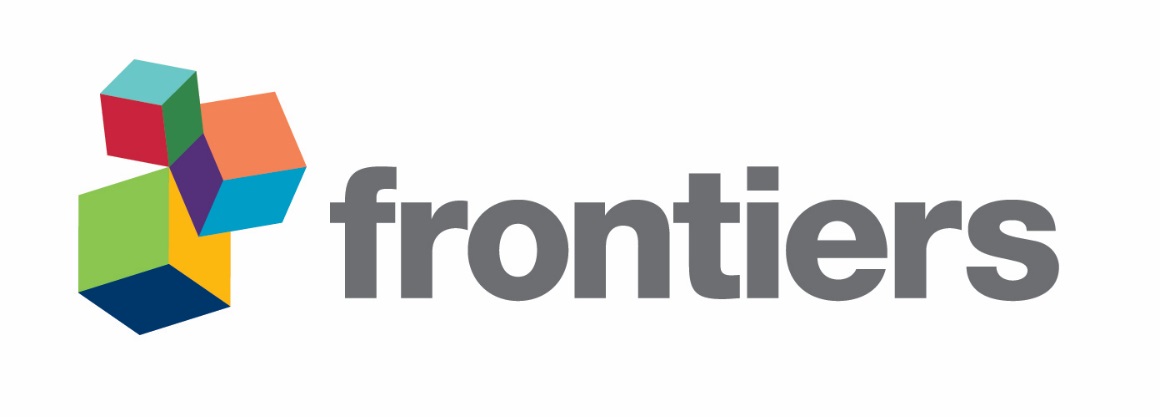
**

**Supplementary Figure 1.** The figure legends are required to have the same font as the main text, 12 point normal Times New Roman, single spaced. Please use a single paragraph for each legend and prepare the figures keeping in mind the PDF layout.
